# The Impact of Tumor Nitric Oxide Production on VEGFA Expression and Tumor Growth in a Zebrafish Rat Glioma Xenograft Model

**DOI:** 10.1371/journal.pone.0120435

**Published:** 2015-03-13

**Authors:** Nadhir Yousfi, Benoist Pruvot, Tatiana Lopez, Lea Magadoux, Nathalie Franche, Laurent Pichon, Françoise Salvadori, Eric Solary, Carmen Garrido, Véronique Laurens, Johanna Chluba

**Affiliations:** 1 INSERM, UMR 866, 'Equipe Labellisée Ligue Contre le Cancer’, Dijon, France; 2 University of Burgundy, UFR SVTE, Dijon, France; 3 EA 7269, Ecole Pratique des Hautes Etudes, Dijon, France; 4 INSERM U1009, Institut Gustave Roussy, Paris, France; University Hospital of Navarra, SPAIN

## Abstract

To investigate the effect of nitric oxide on tumor development, we established a rat tumor xenograft model in zebrafish embryos. The injected tumor cells formed masses in which nitric oxide production could be detected by the use of the cell-permeant DAF-FM-DA (diaminofluorophore 4-amino-5-methylamino-2’-7’-difluorofluorescein diacetate) and DAR-4M-AM (diaminorhodamine-4M). This method revealed that nitric oxide production could be co-localized with the tumor xenograft in 46% of the embryos. In 85% of these embryos, tumors were vascularized and blood vessels were observed on day 4 post injection. Furthermore, we demonstrated by qRT-PCR that the transplanted glioma cells highly expressed *Nos2*, *Vegfa* and *Cyclin D1* mRNA. In the xenografted embryos we also found increased zebrafish *vegfa* expression. Glioma and zebrafish derived *Vegfa* and tumor *Cyclin D1* expression could be down regulated by the nitric oxide scavenger 2-(4-Carboxyphenyl)-4,4,5,5-tetramethylimidazoline-1-oxyl-3-oxide or CPTIO. We conclude that even if there is a heterogeneous nitric oxide production by the xenografted glioma cells that impacts *Vegfa* and *Cyclin D1* expression levels, our results suggest that reduction of nitric oxide levels by nitric oxide scavenging could be an efficient approach to treat glioma.

## Introduction

Transplantation of tumor cells into immunodeficient rodents is commonly used for preclinical drug development in oncology. However, several limitations are inherent to mammalian models, such as, lack of *in vivo* tumor observation, or high costs which impedes high throughput approaches.

During the last decade, zebrafish embryos have become an attractive alternative model for xenografts because they offer many experimental advantages [1,2] and optical transparency of embryos and larvae and adult mutants [3] allows direct *in vivo* imaging of cells. As the specific immune system has not yet developed in two day-old zebrafish embryos, mammalian tumor cells can be easily grafted without rejection in this period. Xenograft models in zebrafish were obtained by injection of rodent and human tumor cells that survived and migrated in embryos, formed tumor masses [4–6], or stimulated angiogenesis [7,8]. Thanks to the transparency of the embryos, tumor cells labeled with fluorescent dyes prior to transplantation enables studies of the interactions between these cells and their environment [9–11]. These models are also useful to evaluate drug effects on cancer [12–15].

Nitric oxide (NO) is a free radical involved in physiological as well as in pathophysiological processes. In tumor biology, NO was demonstrated to promote either tumor invasion and angiogenesis or tumor regression [16–18]. Such ambivalence depends on concentration, timing, nature of cells and enzymes involved in NO production. Studies based on *in vivo* imaging of NO production in tumors should shed more light on these complex processes.

Imaging methods to visualize *in situ* NO production have been described for plant and mammalian cells by using fluorophores [19] such as diaminofluoresceins (DAFs) [20] and diaminorhodamines (DARs) [21]. In the presence of dioxygen, NO induces DAFs transformation into highly fluorescent derivatives. We demonstrated that the use of the cell permeant diaminofluorophore 4-amino-5-methylamino-2’-7’-difluorescein diacetate (DAF-FM-DA) enables to assess *in vivo* NO production in zebrafish embryos [22].

In this study, we analyzed the NO production in glioma xenografts by *in vivo* imaging of DAF labeled zebrafish embryos and performed mRNA expression analysis of *NO synthase* (*Nos*) genes of the tumors and the host. As the xenografts increased in size over time and had vascularized after four days following transplantation, we evaluated the effect of NO on Vascular Endothelial Growth factor-A (*Vegfa*) and *Cyclin D1* gene expression and on tumor growth.

## Materials and Methods

### Cell cultures

The glioma cell line GV1A1 was established from a glioma induced by N-ethyl-N-nitrosourea in a BD-IX rat [23,24]. The cells were grown in RPMI 1640 medium supplemented with 10% (vol) fetal bovine serum (Invitrogen) and Glutamax-I (Invitrogen, Cergy-Pontoise, France).

### Ethical Statement

The zebrafish experiments that are described in the present study were conducted at the University of Burgundy according to the French and European Union guidelines for the handling of laboratory animals. The animal procedures carried out in this study were reviewed and approved by the local Ethics Committee «Comité d’éthique de l’expériment-ation animale Grand Campus Dijon» (C2EA Grand Campus Dijon n°105).

### Zebrafish

Adult wild-type (WIK, ZIRC, Oregon) zebrafish were maintained in a recirculating aquaculture system (Müller & Pfleger, Germany). Photoperiod was 14 L:10D (light:dark), and the mean ranges for conductivity, pH, and temperature in the system were 600–700 μS, 6.0–8.0 and 26–28°C, respectively. The transgenic line Tg (*fli*: EGFP; ZIRC, Oregon) expressing EGFP in endothelial cells was maintained under the same conditions. Staging of zebrafish embryos and larvae was performed as described by Kimmel et al., 1995 [25]. Throughout the manuscript, the age of embryos is indicated as hours post-fertilization (hpf) or days post-fertilization (dpf). Three days before xenotransplantation, females were placed in mating tanks with males. The next morning, mating was initiated by light stimuli and the resulting fertilized eggs were collected. Eggs were collected within one hour of laying and kept at 28° in Petri dishes containing source water (Volvic, France) supplemented with methylene blue (0.3 μg/ml).

### Implantation procedure

Just before transplantation, 48-hpf zebrafish embryos were dechorionized using Pronase (2mg/ml; Roche Diagnostics), anesthetized with tricaine (0.168mg/L) and arrayed on a Petri dish. The glass needles used to inject the cells were made from a glass capillary (1 mm O.D.60.78 mm I.D.; Harvard Appartus) using a PP-830 gravity puller (Narishige). GV1A1 glioma cells were labeled with red or green lipophilic fluorescent tracking dyes (CM-DiI, Invitrogen, or CellTracker Green CMFDA (5-chloromethylfluorescein diacetate; Invitrogen)) before transplantation according to the manufacturer’s instructions. The avascular region of the yolk sac was then injected with a volume of the above-described suspension containing 100–200 cells using the glass needles and the FemtoJet injection system (Eppendorf, Hamburg, Germany). 50 embryos were injected per experimental and control groups. The number of injected cells was counted microscopically by transferring the same volume of injected cells on glass slides using the same glass capillary tubes and injection pressure and duration. The injected embryos were maintained for 1 hour at 28°C prior to incubation at 34°C. At 1 day post injection (dpi), transplanted embryos were examined with a fluorescence stereomicroscope to select embryos containing fluorescent cells. The embryos were then transferred in fresh water (Volvic, France) for further observations. Before microscopy, embryos were anesthetized with tricaine (0.168mg/mL) and after experiments they were euthanized at 6 dpf (or 4 dpi) by an overdose of tricaine (0.3 mg/mL).

### Treatments

Diaminofluorescein-FM-diacetate (DAF-FM-DA) and diaminorhodamine-4M AM (DAR-4M AM) were purchased from Alexis Biochemical (Coger, Paris, France). Embryos were either selected at 1 dpi and transferred into water containing DAF (5μM) or DAR (20μM) and incubated at 34°C for 72 hours, or were selected at 1 dpi and incubated for at least 1 hour with indicated DAF or DAR concentrations at 4 dpi. Embryos were rinsed in water before observation. *In vivo* imaging was performed on anesthetized embryos.

The NO donor glyceryl trinitrate (GTN; Merck, Lyon France) was prepared as a 1% stock solution in ethanol and was directly added to the water at 0.35 μM, 3.5 μM and 35 μM concentrations from 4 dpf to 6 dpf.

The NO scavenger 2-(4-Carboxyphenyl)-4, 4, 5, 5-tetramethylimidazoline-1-oxyl-3-oxide (CPTIO; C221) was purchased from Sigma Aldrich and added to the water (200 μM) after transplantation. The CPTIO containing water was replaced daily until 6 dpf.

### Microscopy

Tricaine anesthetized embryos were put in a small drop of water spotted on a slide and were observed with a Leica MZFLIII fluorescence stereomicroscope bearing appropriate filters. Images of tissue sections were acquired with a Leitz Aristoplan fluorescent microscope coupled to a Leica D-LUX 3LMS camera. *In vivo* images of fluorescent labeled embryos were taken at the CEllImaP platform in Dijon with a Zeiss AxioVert 200M inverted fluorescence microscope (Zeiss, Göttingen, Germany) and appropriate filters (excitation 475/40 nm, emission 530/50 nm) and a cooled charged-coupled device camera controlled with AxioVision software (Zeiss). Live images of tumor cells were collected at the DImaCell platform in Dijon with a Nikon A1-MP biphoton confocal microscope with a 25x objective. For tumor growth observation we used an Axio zoom V16 (Zeiss) macroscope, composed of motorized stereo zoom microscope, a fluorescence light HPX 200 C, coupled to a Zeiss HRm CCD camera and a computer. ZEN software controlled all of the stations and the images were acquired with a 2.3x objective.

For individual tumor development analysis, each xenografted embryo was grown in separate well in 12 well plates. Tumor growth was monitored at 1dpi and 4 dpi by imaging the zebrafish embryos with a Zeiss AxioZoom V16 Macroscope with a constant exposure time. Images were acquired using 2.3x objective and analyzed with ZEN software. For the estimation of tumor size decrease red fluorescence of the tumor surface was measured with ZEN software and data was transferred to Microsoft Excel for further calculations. By this method it is only possible to quantify tumor cell loss as the overall CM-Dil charge remains stable in a growing tumor.

### Histological studies

Xenografted and DAF loaded embryos were fixed in formalin (10%) for 24 h at room temperature after euthanasia with an overdose of tricaine (0.3 mg/mL). A gradual dehydratation in ethanol and xylene was performed. The embryos were embedded in melted paraffin and sectioned at a 5μm thickness. Tissue sections were placed on charged slides, deparaffinised in xylene, and rehydrated through graded alcohol solutions. The sections were then stained with 4', 6-Diamidino-2-phenylindole dihydrochloride (DAPI, Sigma) to visualize cell nuclei.

### Whole-mount alkaline phosphatase staining

Euthanatized embryos were fixed with 4% paraformaldehyde in PBS (pH 7.0) for 2 h at room temperature and then rinsed in PBS. Whole-mount staining of the fixed embryos for endogenous alkaline phosphatase activity was performed according to Nicoli and Presta [7]. Embryos were equilibrated in Tris-HCl (0.1 M; pH 9.5), MgCl_2 (_50 mM), NaCl (0.1 M) and Tween (0.1%) 20 for 30 min and stained with nitroblue tetrazolium (0.4 mg/ml) and X-phosphate (0.1 g/ml; Roche) in the same buffer for 5–15 min. Staining reaction was stopped by adding PBST.

### Reverse-transcription and real-time polymerase chain reaction

Total RNA from pooled embryos (batches of 20 embryos per point per experiment) was isolated with Trizol (Invitrogen, Cergy-Pontoise, France) and reverse transcribed by Moloney murine leukemia virus reverse transcriptase (Promega, Madison, WI, USA) with random hexamers (Promega). Specific forward and reverse primers are summarized in supplementary S1 Table. Real-time quantitative PCR (qRT-PCR) was performed with AmpliTaq Gold polymerase in an Applied Biosystems 7500 Fast thermocycler using the standard SyBr Green detection protocol (Applied Biosystems, Foster City, CA, USA). Briefly, 12 ng of total cDNA, 50 nM (each) primers, and 1x SyBr Green mixture were used in a total volume of 20 μL. All qPCRs were done at least three times in triplicate. Expressions of all genes were normalized respective of rat and zebrafish beta-actin expression levels.

### Embryo dissociation and fluorescence activated cell sorting (FACS)

For FACS analysis of endothelial cells, Tg (fli:EGFP) embryos were dissociated as described previously [26] and analyzed in a on a BD Bioscience LSRII cytometer.

### Data analysis

Results are presented as mean ± SE of triplicates from at least 3 independently performed experiments. Statistical differences between two groups were determined by Student’s t-test and multiple comparisons by ANOVA. P ≤ 0.05 (*) was considered to be statistically significant. NS indicates non-significant differences.

## Results

### Glioma cells grow in zebrafish embryos and produce NO

**Fig 1:**
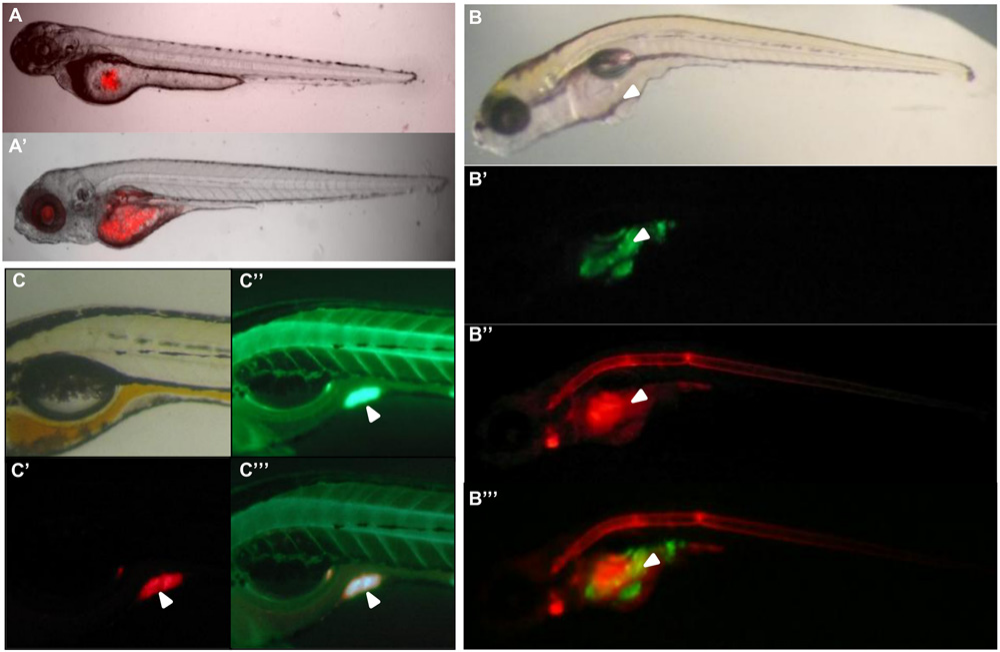
Nitric Oxide detection in xenografted glioma cells. (A) Embryos injected with CM-Dil labeled were cells imaged at 1dpi (A) and 4 dpi (A’). (B) Embryos injected with CellTracker^TM^ Green labeled cells were incubated at 4 dpi with DAR (20μM). The white arrow indicates the outgrowth. (B’) Green labeled glioma cells, (B”) red DAR signal. The specific co-localization is reported in the merge (B”‘). (C) CM-Dil labeled glioma cells transplanted in the yolk and incubated with DAF (5 μM). Specific co-localization is reported in the merge (C”‘). White arrows indicate the tumor area.

Around 100–200 GV1A1 glioma cells labeled with CM-Dil or CellTracker Green were injected into the yolk sac of 2 dpf embryos, which were then observed daily from 1 to 4 dpi to follow tumor formation (S1 Fig). An immune rejection is not possible at this stage as the thymus is not yet functional; according to previous reports, adaptive immunity is not reached before 4 weeks after hatching [27] and only a very small number of T cells could only be observed from 9 dpf [28]. At 1 dpi, the fluorescent cells were detected in the yolk sac and at 4 dpi tumors had significantly grown and formed masses (Fig. 1A).

In order to detect NO production we labeled the embryos with the DAF DAF-FM-DA (4-amino-5-methylamino-2’-7’-difluorescein diacetate), a green fluorescent dye that enables detection of NO production by visualization of a green fluorescent emission. NO production can thus be observed in living Zebrafish embryos by imaging [22]. The fluorophore DAR DAR-4M AM (diaminorhodamine-4M AM) was used to obtain a red label. DAR allowed us to detect NO production by emitted red fluorescence in glioma cells that were labeled with green fluorescent CellTracker Green. Embryos injected with green or red fluorescent labeled glioma cells were incubated with 20μM DAR or 5μM DAF. The signal detected with 20μM DAR is equivalent to that reported with 5μM DAF and generates fluorescent signals in the bulbus arteriosus, notochord, cleithrum and caudal fin [22]. Fluorescence reflecting NO production resulted in a reproducible signal in glioma areas and outgrowths at 4 dpi in the yolk sack (Fig. 1B and 1C; S2A Fig). In some cases, NO production was also detected in tumor environment (Fig. 1B). A case was found where DAF fluorescence formed a fine peritumoral network pattern suggesting NO production by tumor invading blood vessels (S2B Fig).

The percentage of embryos with tumor-NO co-localization was determined. With DAF and DAR, NO production was detected in the tumor areas in 46% of embryos (Table 1). The NO production had no impact on the survival of embryos (Table 1).

**Table 1:** Detection of NO production and survival of rat glioma cell xenografted zebrafish larvae.

	Test	Number of embryos 1 dpi	Number of embryos 4 dpi	Survival (%) 4dpi	Number of larvae with NO production associated to tumor area 4 dpi	NO production and tumor association (%)
DAF 5μM	A	40	21	52.5	9	42.86
	B	40	20	50	11	55
	C	40	11	27.5	5	45.45
	D	40	8	20	3	37.5
	E	40	18	45	8	44.44
	F	40	13	37.14	7	53. 85
			% min	38.69	% min	46.52
			SE	5.3	SE	2,74
DAR 20μM	A	39	18	46.15	8	44.44
	B	44	19	43.18	9	47.37
	C	46	19	41.3	9	47.37
			% min	43.55	% min	46.39
			SE	1.41	SE	0.98

Embryos were incubated at 1 dpi with 5μM DAF-FM-DA or 20μM DAR-4M AM. DAF-FM-DA labelling was carried out 6 times on 40 larvae and DAR-4M AM labelling was done 3 times on 39 to 46 larvae. The embryos were counted again at 4 dpi and NO production was detected by imaging with a Leica MZFLIII fluorescence stereomicroscope.

**Fig 2:**
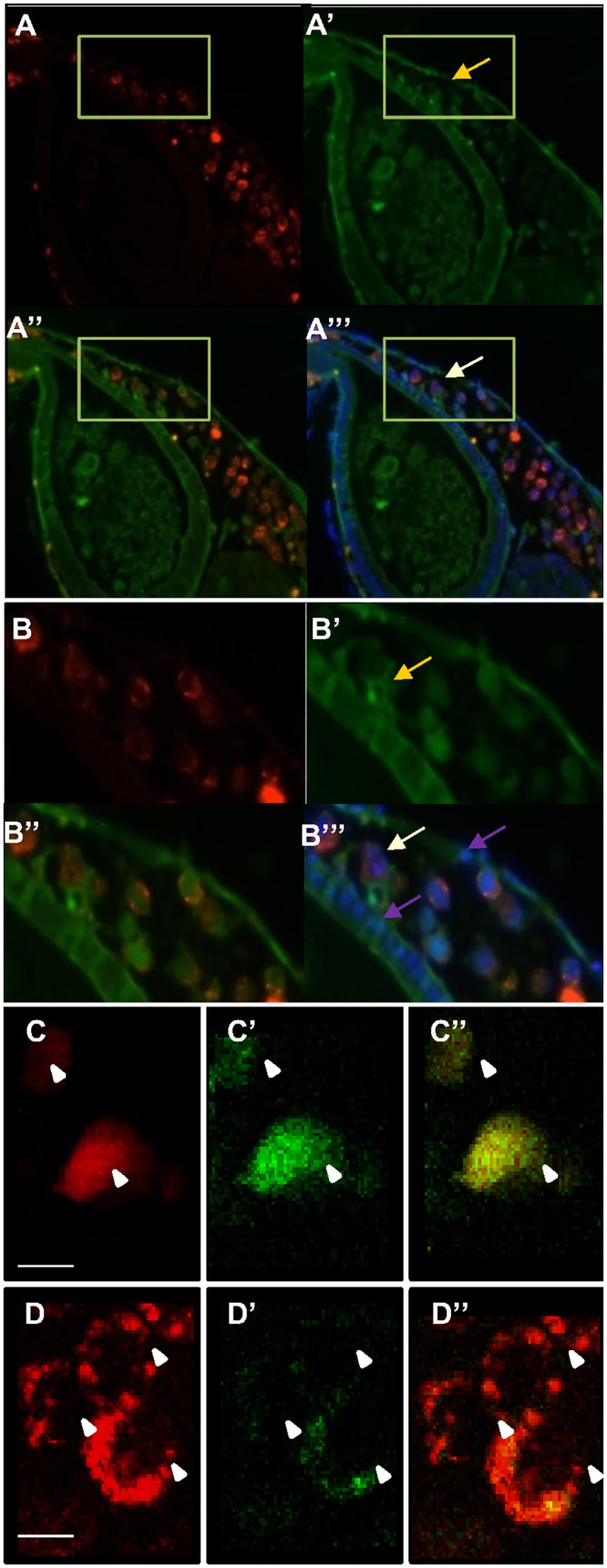
Histology of xenografted tumors and detection of NO in cells. Embryos engrafted with red fluorescent glioma cells and labelled with DAF-FM-DA. Embryos were fixed at 4dpi and sectioned at a 5μm thickness. NO production, detected with DAF signal (A’, A”) was observed in cytoplasm and cell nuclei of tumor cells (yellow arrow). (B) Magnification of yellow framed areas. DAPI labeling was used to detect nuclei (B”‘), enabling detection of the large glioma nuclei and of a dividing tumor cell (white arrow); purple arrows indicate nuclei from zebrafish cells. (C), (D) *In vivo* images of CM-Dil labelled glioma cells taken with a biphoton confocal microscope in 4 dpi DAF treated embryos. White arrows indicate tumor cells. (C) CM-Dil labelled red fluorescent tumor cells, (C”) DAF signal, (C”‘) merge. (D) Red fluorescent glioma cells in DAF and CPTIO treated embryos, (D’) CPTIO decreases DAF fluorescence intensity, showing the specificity of the DAF fluorescence. (D”) merge. White arrows indicate the tumor cells. Scale bars are 10 μM

To identify the nitric oxide producing cells sections of xenografted DAF labeled embryos (Fig. 2A and 2B) were performed. The DAF signal is conserved in histological sections, so it could be observed in cytoplasm and in the nucleus of tumor cells as described previously by Saini *et al*. [29]. Tumor cells were detected by red fluorescent cell tracker spot signals in perinuclear vesicles and large DAPI labeled nuclei. The size difference of the nuclei allowed us to distinguish rat glioma cells from zebrafish cells (Fig. 2B). In addition, DAPI labeling revealed a dividing tumor cell showing that the cells proliferate at 4 dpi (Fig. 2B). Live imaging of 4 dpi xenografted embryos with a biphoton confocal microscope confirmed NO in rat glioma cells (Fig. 2C and 2D). This DAF signal is specific as it disappeared after treatment with the NO scavenger CPTIO (Fig. 2D).

**Fig 3:**
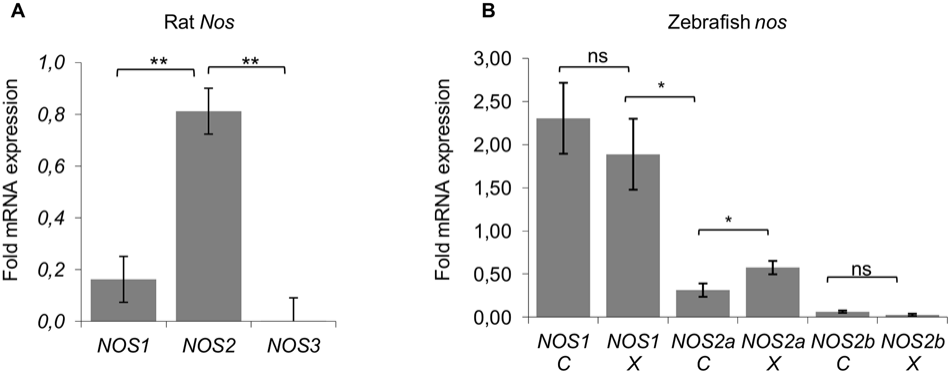
QRT-PCR analysis of *nos* gene expression in control and xenografted embryos. (A) In xenografted embryos, rat *Nos2* is highly expressed in comparison to *Nos1* and *Nos*3. (B) Zebrafish *nos1* gene is strongly expressed in transplanted and control embryos. A significant difference is only observed for *nos2a*. Error bars represent SE, * p≤ 0, 05, **<0,01 (one way ANOVA).

NO is catalyzed from L-arginine by nitric oxide synthase (NOS) enzymes, which have been identified in vertebrates, invertebrates and bacteria. Three isozymes exist in mammals: NOS1, NOS2 and NOS3 [30], and in zebrafish, NOS1, NOS2a and NOS2b [31–33]. In order to determine which *Nos* are expressed by the glioma cells *in vitro* and which are induced by the xenografts in zebrafish we performed qRT-PCR analyses. *Nos2* was clearly overexpressed in the xenografted glioma cells when compared to *Nos1* and *Nos3* (Fig. 3A). In contrast, *Nos2* expression in cultured glioma cells depended on the cell density and was very variable (not shown). In zebrafish, *nos1* was the highest expressed NO synthase gene in both, control and xenografted embryos; there was no significant difference between control and xenografted groups. The same was the case for *nos2b*. However, for *nos2a*, the *nos* isoform that corresponds to mammalian *Nos2*, a significant increase was observed in the xenografted embryos (Fig. 3B).

### Effect of NO production on angiogenesis

**Fig 4:**
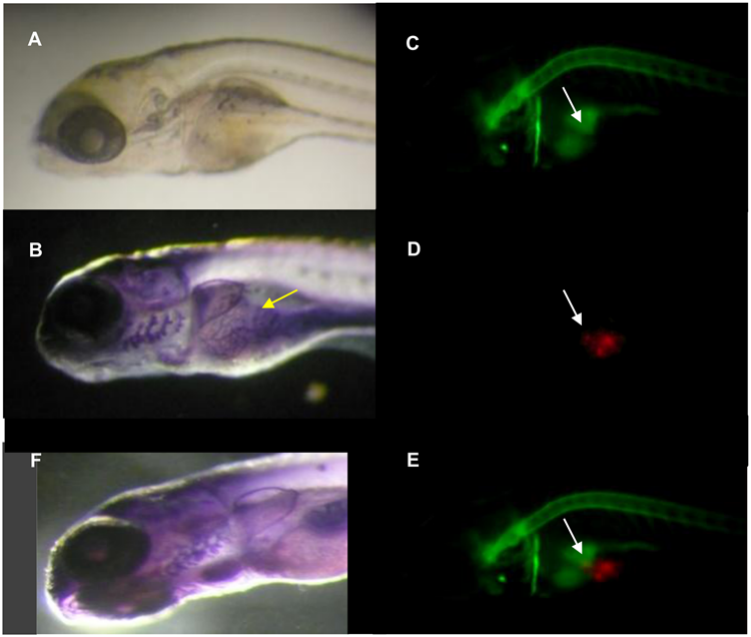
Alkaline phosphatase assay of xenografted DAF-FM-DA treated embryos. Embryos were injected with red CM-Dil labeled tumor cells, incubated at 4 dpi in DAF (5μM) and fixed at 4dpi before detection of alkaline phosphatase activity. (A) Bright field image of the embryos before treatments. (B) Neovascularisation was observed in 12 of 14 (85.5%) embryos presenting NO production in tumor environment. (D) Fluorescence detection of glioma cells, (C) DAF signal, (E) merge. The white arrows indicate the part of the tumor mass that co-localizes with DAF fluorescence signal. (F) A control embryo with absence of vessels in this region of the yolk.

NO is a key mediator in angiogenesis and tumor progression, but is also known to promote or inhibit vessel formation depending on concentration and duration of exposure [16]. We analyzed blood vessel formation using alkaline phosphatase labeling [7] in xenografted embryos in which we detected tumor NO production. Neovascularisation in close proximity of the tumors was observed in 85% (12/14) of the embryos presenting NO production in the tumor area (Fig. 4B). In these embryos, the network profile of phosphatase alkaline detection overlapped with the NO production profile obtained with DAF labeling (Fig. 4C and 4E) and the glioma mass (Fig. 4D and 4E). Overlap of CM-Dil and DAF signal is only partial and indicated by the arrow in Fig. 4E. In non-injected embryos, there are no vessels in this region, as reported earlier by Nicoli et al. 2007 [34], also shown in the control embryo (Fig. 4F).

**Fig 5:**
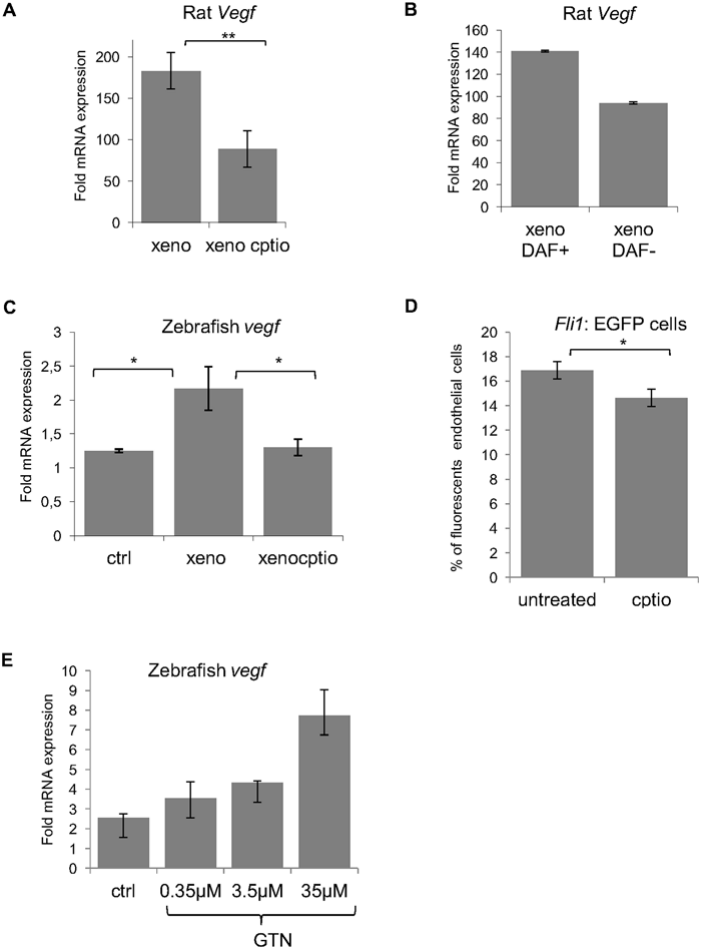
QRT-PCR analysis of *Vegfa* expression in control and xenografted embryos. (A) Glioma cell *Vegfa* expression significantly decreases in presence of the NO scavenger CPTIO. Error bars represent SE, **<0, 01, Studtent’s t-test. The same effect was observed for zebrafish *vegfaa* (C), compared to non xenografted embryos and CPTIO treatment. Error bars represent SE, * *p* ≤ 0, 05 (one way ANOVA). (B) Embryos were stained with DAF-FM-DA at 4 dpi and separated in DAF signal positive and negative groups. *Vegfa* mRNA level is higher in the DAF positive group. One representative experiment from three independent experiments is shown. (D) Tg (*fli*: EGFP) CPTIO treated embryos that did not receive xenograft were lysed and endothelial cells sorted by FACS. The bars represent the mean percentage of sorted endothelial cells in presence and absence of CPTIO. Error bars represent SE, * *p* < 0, 05, Student’s t-test. (E) Zebrafish embryos were incubated with increasing concentrations of GTN. Expression of zebrafish *vegfaa* is dose dependent at the indicated concentrations. One representative experiment from three independent experiments is shown.

In order to find a correlation between NO production and angiogenesis, we measured the mRNA levels of rat *Vegfa* and zebrafish *vegfa* in the presence and absence of CPTIO, a NO scavenger. QRT-PCR revealed a decrease of glioma *Vegfa* expression (Fig. 5A). The link between NO production and *Vegfa* expression was verified by comparing DAF-positive and DAF-negative tumors. DAF negative tumors expressed less *Vegfa* than the NO producing tumors (Fig. 5B). Two *vegfa* genes exist in zebrafish: *vegfaa* and *vegfab* [35]. Zebrafish *vegf*aa levels also decreased in presence of the CPTIO (Fig. 5C). As the expression of *vegfab* remained unchanged in xenografted embryos, results are not shown.

The effect of NO on endothelial cells was confirmed by incubation of non-xenografted embryos of the transgenic line Tg (*fli*:*EGFP*) in 200μM of CPTIO from 2 dpf to 6 dpf. At 6 dpf, the embryos were dissociated and the number of endothelial cells were quantified by FACS analysis. In fact, a significant decrease in endothelial cell numbers was observed when embryos were treated with CPTIO compared to controls (Fig. 5D). This result correlates with the increase of zebrafish *vegfaa* expression when non-xenografted embryos were treated with increasing concentrations of the nitric oxide donor GTN (Fig. 5E).

### NO and tumor growth

**Fig 6:**
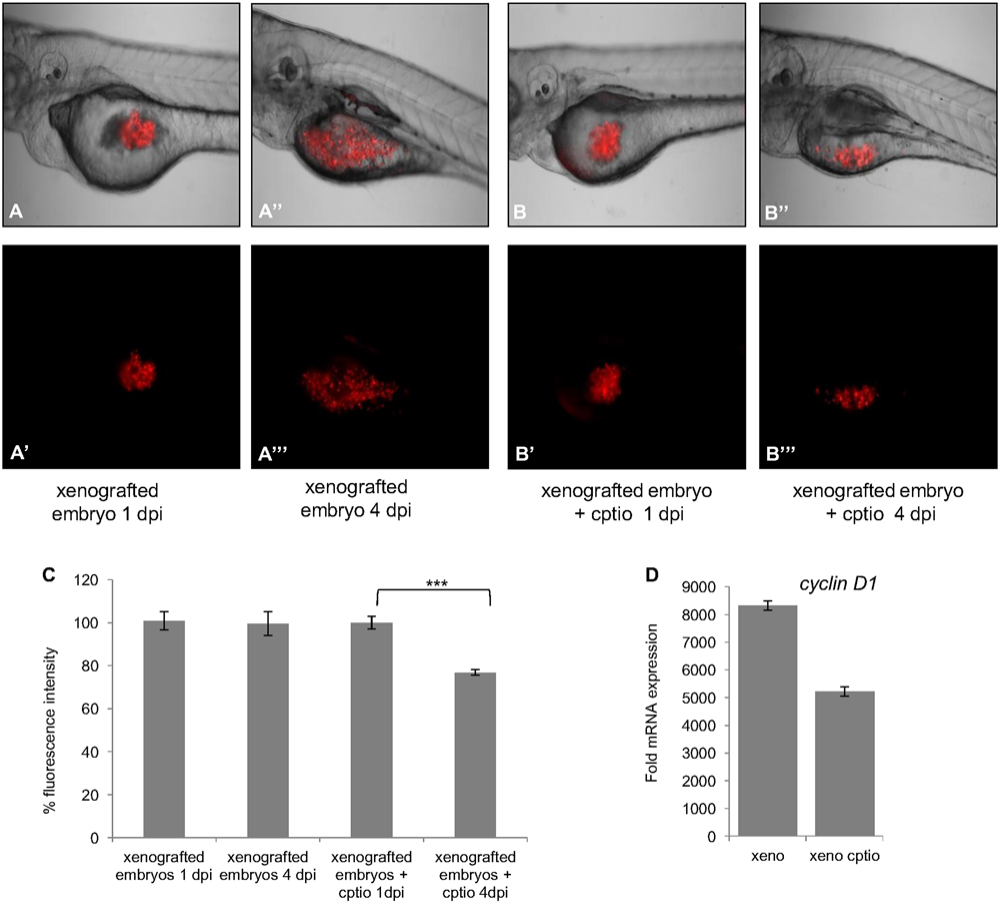
The effect of NO on tumor growth. CM-Dil labeled glioma cells were injected into the yolk of zebrafish embryos at 2 dpf and incubated with CPTIO (200μM). Images were taken at 1 dpi and 4 dpi. Images (A-A”‘) and (B-B”‘) show the tumors at 1 dpi and 4 dpi for control and CPTIO treated embryo. At 4 dpi, tumors have increased in size in untreated embryos (A”-A”‘) in comparison to CPTIO treated embryos (B”-B”‘). (C) Percentage of fluorescence intensity of the tumor surfaces was determined by ZEN software (n = 10). Images were taken with an Axio zoom V16 (Zeiss) macroscope, with the same exposure time for all embryos. Error bars represent SEM, *** *p* <0, 001, (one way ANOVA). (D) QRT-PCR analysis of *Cyclin D1* gene expression in untreated and CPTIO treated xenografted embryos. *Cyclin D1* expression was decreased upon CPTIO treatment. One representative experiment from four independent experiments is shown.

We assessed the effect of NO on tumor growth by imaging untreated and CPTIO treated xenografted embryos and by comparing tumor size at 1 dpi and 4 dpi in both groups. At 4 dpi, in the untreated group tumors have grown with local invasion of the yolk (Fig. 6A), whereas in the CPTIO group, the tumor size decreased (Fig. 6B). Moreover, at 4 dpi the tumors of CPTIO treated embryos displayed 20% lower fluorescence intensity per tumor surface than those of the control group (Fig. 6C), which is a sign of a loss of tumor cells.

Cyclin D1 regulates the transition from the G1 phase to the S phase of the cell cycle. Amplification or over-expression of *Cyclin D1* plays a pivotal role in the development of a subset of human cancers, including those of the breast, liver, retina and esophagus [36,37]. The expression of rat *Cyclin D1* was analyzed in the xenografts by qRT-PCR, in order to see if there is a correlation with NO production. In fact, a decrease of rat *Cyclin D1* expression was noted in CPTIO treated xenografted embryos when compared to the control (Fig. 6D). This indicates that the glioma cell proliferation is hampered by the absence of nitric oxide.

## Discussion

There is increasing evidence that NO plays a role in tumor angiogenesis, vascular functions, tumor progression and metastasis [16–18]. Most of the *in vivo* experimentations performed so far were carried out in mammalian models or *in vitro* where it is difficult to assess the importance of tumor environment, space repartition and dynamics of NO production along tumor growth [38].

A BD-IX rat glioma xenograft model was developed in zebrafish to study tumor NO production and its effects *in vivo*. When injected in the yolk sac, tumor cells formed masses at the injection site. We show that the NO specific probes DAF-FM-DA and DAR-4M-AM revealed NO production in tumor areas and inside tumor cells. NO could be co-localized with the tumor cells as shown by histological and biphoton confocal analysis (Fig. 2). NO could also be detected in regions near tumors, and in one case DAF fluorescence had a vessel-like pattern, suggesting the presence of NO in the blood vessels (S2B Fig). However, only 45% of tumors or parts of tumors were positive for NO. This could be explained by cellular heterogeneity in this mixed rat glioma line [23] or shear stress that has been reported to able to induce *NOS2* expression[39,40].

NO is produced by specific enzymes, the NOS family. The rat G1A1 glioma cell line used in this study expressed *Nos2* and low levels of the other *Nos*. While *Nos2* expression was highly variable *in vitro*, it was over-expressed and stable in the xenografts. When we analyzed zebrafish *nos* expression in whole embryos injected with the glioma cells, we did not detect any significant increase in zebrafish *nos1* and *nos2b* isoforms but saw an increase of *nos2a levels*, suggesting an effect of tumor-produced NO on zebrafish cells.

In several earlier reports, tumor xenografts have been shown to be vascularized in zebrafish [8,34,41]. Angiogenesis was also detected in a large proportion of the zebrafish embryos displaying NO production in the xenografted gliomas, indicating that our model allows the exploration of the relationships between these events [42]. Tumor angiogenesis has been shown to be induced by tumor-derived VEGFA production in zebrafish [11,34,43,44]. Our data confirms rat *Vegfa* expression in the xenografts but also show increase of *vegfaa* expression in the xenografted embryos. The significant decrease of glioma-derived and zebrafish *Vegfa* expression in the presence of CPTIO confirmed this NO link with angiogenesis. In addition, CPTIO treatment decreased the number of endothelial cells of non-xenografted zebrafish embryos.

Finally, we show that NO scavenging by CPTIO has an effect on tumor growth. When the xenografted embryos were treated with CPTIO, the tumor fluorescence intensity was decreased by 20% compared to the controls at 4 dpi. This decrease can be explained by a loss of cells, as the control group did not change in CM-Dil fluorescence intensity. This result is supported by a decrease of *Cyclin D1* expression under CPTIO treatment. This data confirms an earlier report showing that total inhibition of NO by scavengers reduced tumor vascularization and tumor growth [45].

In conclusion, we demonstrate that glioma cell xenograft model in zebrafish enables *in vivo* imaging of tumor NO production and the study of NO effects on tumor growth. This model may be useful for preclinical development of anti-angiogenic or other antitumor drugs targeting NO.

## Supporting Information

S1 Fig Xenotransplantation procedure. DAF: DAF-FM-DA, DAR: DAR-4M AM, PA: Alkaline phosphatase assay.
(TIFF)

S2 Fig Images of nitric oxide detection in xenografted embryos. Embryos were injected with CM-Dil labeled glioma cells. At 4dpi DAF (5μM) was added to the water. After rinsing embryos were imaged with an inverted fluorescence microscope. (A) Bright field image of a xenografted embryo at 4 dpi; (A’) CM-Dil labeled tumor cells; (A”) DAF label; (A”‘) merge. The white arrows indicate tumor mass. An embryo with a different DAF-FM-DA pattern is shown in (B). Glioma cells are red, DAF signal appears green. (B) glioma cells; (B’) magnification from (B); (B”) DAF signal; (B”‘) magnification of the merge. The white arrows indicate tumor cells, the yellow arrows the network-like DAF fluorescence pattern resembling blood vessels.
(TIFF)

S1 Table Primers used for qRT-PCR. (PDF)
