# Supplementary figures and images for: The Impact of Tumor Nitric Oxide Production on VEGFA Expression and Tumor Growth in a Zebrafish Rat Glioma Xenograft Model
Source: PLoS One. 2015 Mar 13;10(3):e0120435. doi: 10.1371/journal.pone.0120435 (PMC4359111; doi:10.1371/journal.pone.0120435)

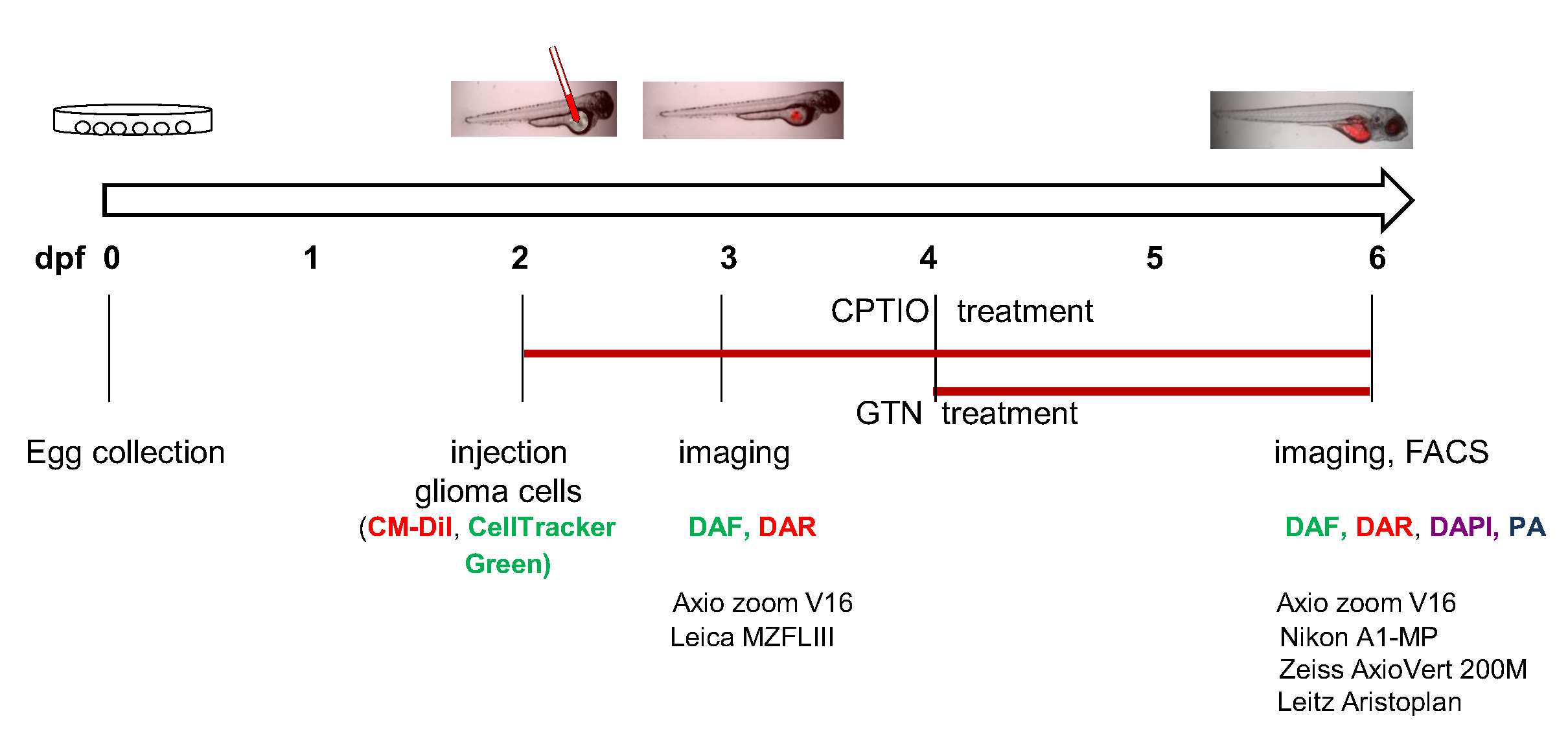

Supplement: S1 Fig — DAF: DAF-FM-DA, DAR: DAR-4M AM, PA: Alkaline phosphatase assay. (TIFF) [file pone.0120435.s001.TIFF]

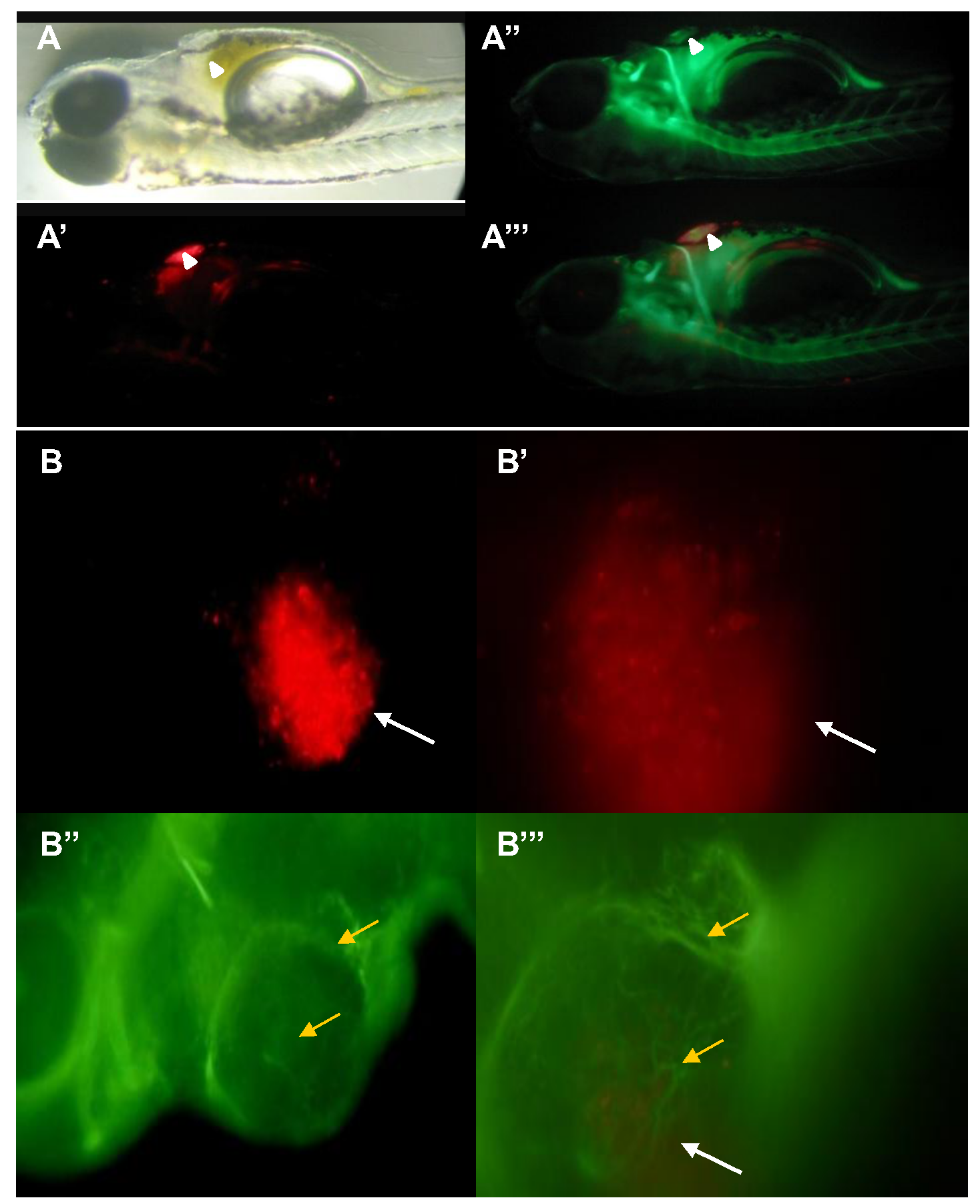

Supplement: S2 Fig — Embryos were injected with CM-Dil labeled glioma cells. At 4dpi DAF (5μM) was added to the water. After rinsing embryos were imaged with an inverted fluorescence microscope. (A) Bright field image of a xenografted embryo at 4 dpi; (A’) CM-Dil labeled tumor cells; (A”) DAF label; (A”‘) merge. The white arrows indicate tumor mass. An embryo with a different DAF-FM-DA pattern is shown in (B). Glioma cells are red, DAF signal appears green. (B) glioma cells; (B’) magnification from (B); (B”) DAF signal; (B”‘) magnification of the merge. The white arrows indicate tumor cells, the yellow arrows the network-like DAF fluorescence pattern resembling blood vessels. (TIFF) [file pone.0120435.s002.TIFF]
