# Supplementary material for: The Impact of Tumor Nitric Oxide Production on VEGFA Expression and Tumor Growth in a Zebrafish Rat Glioma Xenograft Model
Source: PLoS One. 2015 Mar 13;10(3):e0120435. doi: 10.1371/journal.pone.0120435 (PMC4359111; doi:10.1371/journal.pone.0120435)
Supplement: S1 Table — (PDF) [file pone.0120435.s003.pdf]

S1 Table 1. Primers used for qRT-PCR

Gene name	Primers	PCR product lenght	Accession number
**Rat genes**			
Actb	Fw: ACCCGCGAGTACAACCTTC<br>Rv: GCAGCGATATCGTCATCCA	83 bp	NM_031144.3
Vegfa	Fw: ACCAGCGCAGCTATTGCCGT<br>Rv: AGGGGCACACAGGACGGCTT	100 bp	NM_001110333.2
Cyclin D1	Fw: AAGGTTTAGGGCCATGTCTG<br>Rv: GGAGACCACATCTCCGTCTT	96 bp	NM_171992.4
Nos1	Fw: GACAACGTTCCTGTGGTCC<br>Rv: CCGTTCTTGGTGGGAGAC	86 bp	NM_052799.1
Nos2	Fw: CTCTCAGCAGCATCCACG<br>Rv: GCGGCTGGACTTCTCACT	83 bp	NM_012611.3
Nos3	Fw: CCGAGGCAATCTTCGTTC<br>Rv: GCTGGCTGTTCCAGATCC	83 bp	NM_021838.2
**Zebrafish genes**			
actb	Fw: CCCAGACATCAGGGAGTGAT<br>Rv: CACAATACCGTGCTCAATGG	117 bp	NM_131031.1
vegfaa	Fw: TGTCGGGACGCATTCGAGCG<br>Rv: CGAGCACACACGTACATCCCGTCC	87 bp	NM_001110349.2
vegfab	Fw: TGCTGTTCGCGTGCTCCAGT<br>Rv: GCTTCTGCCTCCCTCTCTGGGT	91 bp	NM_001044855.2
nos1	Fw: GAAAGAGATCGAGCCTGTGC<br>Rv: TCCTATCGATGACCCTCTGG	83 bp	NM_131660.1
nos2a	Fw: TGTCTTTGGCCTGGGTTC<br>Rv: GCTCCGCAAGCTTGTCAT	77 bp	NM_001104937.1
nos2b	Fw: TTGGTCTTGGCTCTCGGA<br>Rv: GATGGCTCCCAGTGTTGC	83 bp	NM_001113501.1
